# P-1400. Development of a community advisory group for TB research

**DOI:** 10.1093/ofid/ofaf695.1587

**Published:** 2026-01-11

**Authors:** Padmasayee Papineni

**Affiliations:** London North West University Healthcare NHS Trust, London, England, United Kingdom

## Abstract

**Background:**

North West London has one of the highest tuberculosis (TB) rates in the UK. A number of clinical trials have used community advisory boards (CAB) that include affected persons, members of community based organisations and health advocates, however there are limited UK-based patient groups that are representative of affected populations who can advise on TB research.

**Figure:**
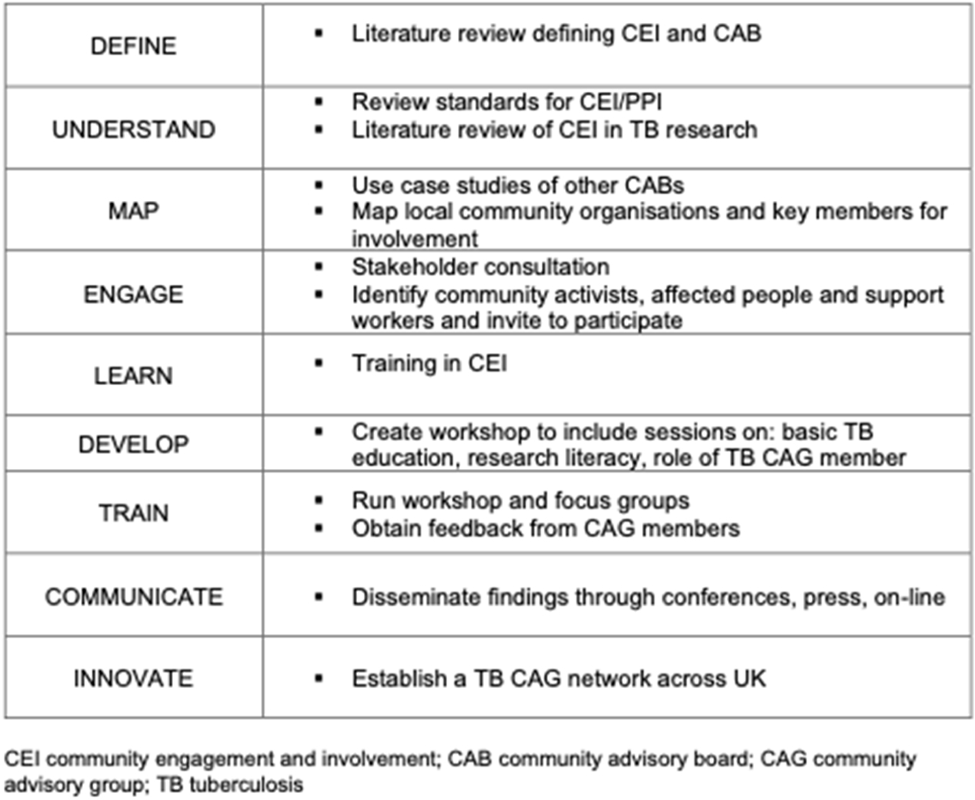
Methods and objectives in development of a community advisory group for TB research

**Methods:**

Used evidence from community engagement from TB clinical trials and research initiatives of practical recommendations, created a basic TB education resource and designed a research literacy workshop.

**Results:**

There were a total of 9 participants ages ranged from 27 -55 years with an average of 40.5 years; 66% were female. The majority (6) of participants were of Indian ethnic background. Most participants had attended university (8) with one having a professional qualification. Two participants had lived experience of TB, and are currently receiving treatment for TB disease.

There was good awareness of TB symptoms, duration of treatment and factors that can cause drug resistance. *TB-CAG knowledge of research and community engagement* was an understanding that it was about finding answers to questions and gaining deeper understanding of disease and health. Participants highlighted that community meant sharing certain characteristics, and gave examples of religious and living in a geographical location. Themes from the group discussion was around delays in diagnosis, stigma, fair representation on the CAG and how the CAG could be used for TB awareness/education.

**Conclusion:**

This project was a pilot of creating a training workshop for a TB community advisory group that represents our local population. The CAG created is diverse and the feedback from the sessions is positive that the participants want to have agency and are keen to provide a community voice to TB research. The next steps include: *Inclusion*: Train more CAG members, including those with lived experience of drug resistant TB, latent TB and parents caring for children with TB and women who have experience TB in pregnancy. *Dissemination* presentation to networks; deliver education sessions at temple, mosque, gurdwara. *Partnerships* Develop a network across the UK of a national TB community advisory group.

**Disclosures:**

All Authors: No reported disclosures

